# Spatial Expression Pattern of the Major Ca^2+^-Buffer Proteins in Mouse Retinal Ganglion Cells

**DOI:** 10.3390/cells9040792

**Published:** 2020-03-25

**Authors:** Tamás Kovács-Öller, Gergely Szarka, Ádám J. Tengölics, Alma Ganczer, Boglárka Balogh, Edina Szabó-Meleg, Miklós Nyitrai, Béla Völgyi

**Affiliations:** 1 János Szentágothai Research Centre, University of Pécs, 7624 Pécs, Hungary; gergely.sz@gmail.com (G.S.); alma.ganczer@gmail.com (A.G.); boglarka.balogh311@gmail.com (B.B.); edina.meleg@aok.pte.hu (E.S.-M.); miklos.nyitrai@aok.pte.hu (M.N.); volgyi01@gamma.ttk.pte.hu (B.V.); 2 Retinal Electrical Synapses Research Group, National Brain Research Program (NAP 2.0), Hungarian Academy of Sciences, 1051 Budapest, Hungary; 3 Department of Experimental Zoology and Neurobiology, University of Pécs, 7624 Pécs, Hungary; 4 Medical School, University of Pécs, 7624 Pécs, Hungary

**Keywords:** parvalbumin, calretinin, calbindin, expression, retina, topography, ganglion cell, calcium buffer protein, expression

## Abstract

The most prevalent Ca^2+^-buffer proteins (CaBPs: parvalbumin—PV; calbindin—CaB; calretinin—CaR) are widely expressed by various neurons throughout the brain, including the retinal ganglion cells (RGCs). Even though their retinal expression has been extensively studied, a coherent assessment of topographical variations is missing. To examine this, we performed immunohistochemistry (IHC) in mouse retinas. We found variability in the expression levels and cell numbers for CaR, with stronger and more numerous labels in the dorso-central area. CaBP+ cells contributed to RGCs with all soma sizes, indicating heterogeneity. We separated four to nine RGC clusters in each area based on expression levels and soma sizes. Besides the overall high variety in cluster number and size, the peripheral half of the temporal retina showed the greatest cluster number, indicating a better separation of RGC subtypes there. Multiple labels showed that 39% of the RGCs showed positivity for a single CaBP, 30% expressed two CaBPs, 25% showed no CaBP expression, and 6% expressed all three proteins. Finally, we observed an inverse relation between CaB and CaR expression levels in CaB/CaR dual- and CaB/CaR/PV triple-labeled RGCs, suggesting a mutual complementary function.

## 1. Introduction

Vision is the most important perception modality for humans, however, it is still not entirely known how the Ca^2+^ homeostasis of neurons in the visual signaling axis is controlled by the various neuronal Ca^2+^-buffer proteins (CaBPs). One of the most extensively studied brain loci in this regard is the mammalian retina where expression of CaBPs has been described in many different neuron populations of various model animals [1] and also in a human specimen [2]. Retinal ganglion cells (RGCs) serve as the only output of the retina, and besides simple integration of the converging inputs, they preprocess information prior to relaying it to higher visual brain centers. Therefore, RGCs play a key role in visual signal processing and thus the knowledge of the molecular makeup underlying RGC function is essential both for further scientific endeavors and clinical diagnostics. The RGC population in the mammalian retina is a collection of neurons encoding several different visual features of the surrounding world. The diverse coding mechanism each RGC subtype performs is heavily determined by a unique composition of intracellular molecules. Some of these molecules, like CaBPs, can also be utilized as markers for one or multiple distinct cell groups. The more-than 30 RGC subtypes of the mouse retina [3,4] are currently in the crosshairs of modern visual neuroscience and their CaBP expression patterns are in the focus of mainstream research.

CaBPs can bind multiple Ca^2+^s, therefore, having a central role in Ca^2+^ maintenance. They can control intracellular Ca^2+^-dependent signal transduction by actively buffering Ca^2+^ levels. Some of them, like calmodulin, can be direct partners for other proteins taking part in signal transduction (Ca^2+^/calmodulin-dependent protein kinase in this case), allowing them to function as Ca^2+^ sensors as well [5].

We focused on the three most abundantly expressed CaBPs in RGCs, namely parvalbumin (PV), calbindin (CaB), and calretinin (CaR). PV is the smallest of the three in size (only ~12 kDa), with two functional Ca^2+^-binding sites and three EF-hand motifs (helix-loop-helix motif) [6]. CaB is a bigger (28 kDa), globular-shaped protein with two Ca^2+^-binding sites and six EF-hand domains [7]. CaR is a 29-kDa-size protein and it possesses five functional Ca^2+^-binding EF-hand domains and an additional non-functional one [6]. CaR also shares significant structural similarity with CaB which caused scientists to first identify it as ‘calbindin-2′ (for detailed structural and 3D PDB data, see: https://www.rcsb.org [8]).

Parvalbumin (PV) is one of the three most studied and best known Ca^2+^-binding buffer proteins and it has been shown to be expressed by eight different mouse RGC subtypes [9,10]. Calretinin (CaR) and calbindin (CaB) expressing RGCs have also been characterized [11,12], among which five populations were shown to co-express these two proteins. There is sparse evidence for a circadian and/or light adaptation-dependent modulation of expression levels of the above three Ca^2+^-buffer proteins: only PV has been shown to have diurnal/light adaptation changes in the retina [13], although calbindin is known to be expressed by interneurons in the suprachiasmatic nucleus [14], which take part in circadian regulation. Additionally, there is no evidence for any adaptational changes in the retina regarding CaB and CaR. Due to the fact that Ca^2+^ buffering is strongly related to the kinetics of the signal (action potential train) RGCs send towards the brain, it is possible that individual RGCs of the same subtype might show a range of CaBP expression levels that induces subtle changes in intracellular Ca^2+^ transients [15] and correlates with the activity of the respective RGC or their topographical location. Many previous studies have used these and the less widely expressed CaBPs as a means to cell identification in the retina (reviewed in detail by Kovacs-Oller et al. [1]). Considering the CaBPs evaluated in this study, CaB can be utilized as a marker for horizontal cells (HC) as they are expressed by axon-bearing HCs in a wide range of species, including the mouse [16]. CaR and PV are both considered as an AII amacrine cell marker in several species [17,18]. The mouse retina is somewhat of an exception in this regard, since AII cells appear negative for PV labels [19]. Even though CaBPs have served as a useful tool for cell identification in histological studies, there is currently no substantial data available considering CaBP functional and/or expressional interchangeability, meaning that co-expression studies could provide the first step towards the understanding of how these proteins regulate Ca^2+^ levels and neuronal function.

Here, we examine how the expression levels of the three major CaBPs differ and also how levels of each of these proteins change across the retina and whether they display any topographically modulated pattern.

In this study, we present PV, CaR, and CaB expression in the same subset of neurons for the first time, which allows a direct comparison of the expression levels of the three CaBPs in mouse retinal RGCs. We found heterogeneity in the expression levels and the number of expressing cells for CaR with stronger and more numerous labels in the dorso-central area. The contribution of labeled cells to the total cell count was homogenous for both CaB and CaR, whereas PV expressing cells comprise about half of the RGCs in the center but only a quarter of peripheral RGCs. CaBP expressing cells contributed to RGCs with all soma sizes, indicating the heterogeneous nature of CaB, CaR, and PV expression across various RGC subtypes. We separated four to nine RGC clusters in each retinal area based on CaBP expression levels and soma sizes. Besides the overall high variety in the number and size of clusters, the peripheral half of the temporal retinal quadrant showed the highest number, possibly indicating a better separation of functional RGC subtypes in this area. In addition, our multiple labeling experiments showed that 39% of the RGCs showed positivity for a single CaBP, 30% expressed two CaBPs, 25% showed no CaBP expression, and 6% expressed all three proteins. Finally, we observed an inverse relation of the CaB and CaR expression levels in CaB/CaR dual- labeled and CaB/CaR/PV triple-labeled RGCs, suggesting a potential complementary function of these two CaBPs.

## 2. Materials and Methods

### 2.1. Animals and Preparation

Animal handling, housing, and experimental procedures were reviewed and approved by the ethical committee of the University of Pécs (BA02/2000-6/2006). All animals were treated in accordance with the Association for Research in Vision and Ophthalmology, Statement for the Use of Animals in Ophthalmic and Vision Research. All efforts were made to minimize pain and discomfort during the experiments. Mice were deeply anesthetized with the inhalation of Forane (4%, 0.2 mL/L) and then sacrificed using cervical dislocation. The mice’s eyes (*Mus musculus*, C57BL/6J, 1–12 months, n = 10 animals, male; PV-Cre (JAX #017320) x tdTomato (JAX #007909) n=3, male) were removed immediately after termination. Eyeballs were cut at the ora serrata, then the lens and vitreous body were removed. Retinas were fixed in 4% paraformaldehyde in 1× PBS at room temperature for 15 min (for details, see: [20]).

### 2.2. Immunohistochemistry and Microscopy

**Figure 1:**
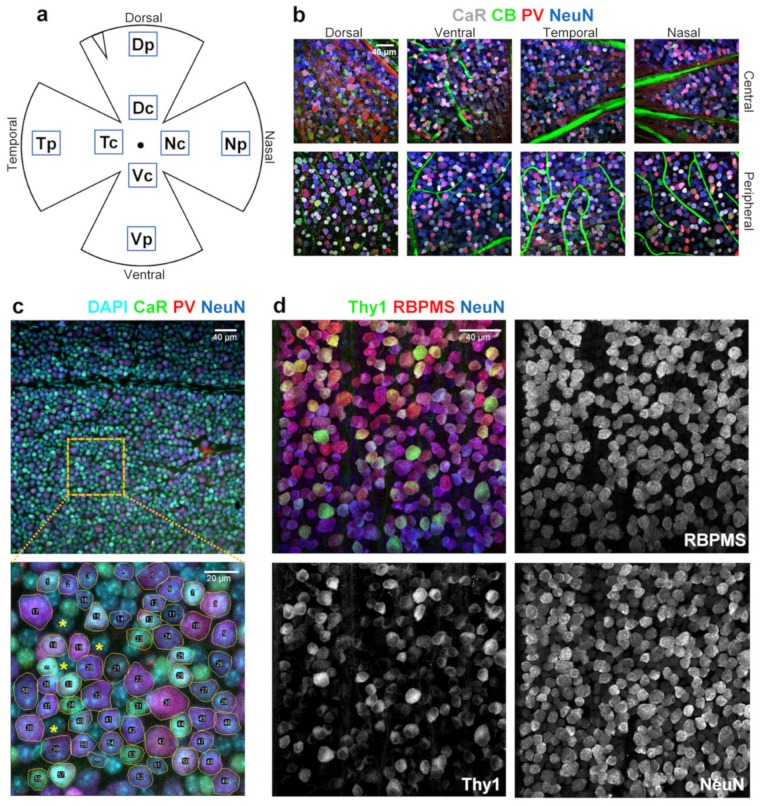
Measurement of Ca^2+^-buffer protein (CaBP) expression levels in retinal samples. (**a**) Schematic drawing of approximate locations of selected retinal areas (V—Ventral, N—nasal, D—Dorsal, T—Temporal; c—central, p—peripheral). (**b**) Representative retinal areas with all utilized cellular markers. (**c**, **d**) Whereas DAPI (4′,6-diamidino-2-phenylindole) labels all cellular nuclei and therefore marks both neuronal and non-neuronal cells simultaneously, neuronal nuclei antibody (NeuN) stains neurons exclusively. In addition, NeuN provides brighter staining for retinal ganglion cells (RGCs), while at the same time, displaced amacrine cells (ACs) appear dimmer, this distinction is visualized using RBPMS antibody (RNA-binding protein with multiple splicing) staining on a GCaMP3-Thy1 (THYmocyte differentiation antigen 1) mouse retina. This provides a clear basis for separating ganglion cells (GCs) from displaced ACs, allowing us to disregard ACs in our analysis. A central 100 × 100 μm homogeneous sub-area was selected from each 20× scanned and merged stack to measure the expression levels of the three CaBPs. All NeuN+ cells were measured for calretinin (CaR), parvalbumin (PV), and calbindin (CB) expression levels (only triple and quadruple labelings were included in the measurements). Cells were numbered using FIJI’s regions of interest (ROI) manager.

Following a thorough washing in PBS, the dorsal aspect and topographical areas (see Figure 1a) (using the choroid marks [21]) were clearly marked. Next, the retinas were isolated (Figure 1a). Blocking was performed with BTA (bovine serum albumin 5%, Triton X-100 0.5%, Sodium-azide 0.05% in PBS) on the bottom of a 24-well plate. Primary antibodies were used as indicated in Table 1. After washing (×3) with PBS, secondary antibodies were added (Table 1). Retinas were mounted with VectaShield (vector and nr.1 cover slides) and inspected using a Zeiss LSM 710 confocal laser scanning microscope (PlanApochromat 20× and 63× objectives; NA: 1.4; Carl Zeiss Inc., Jena, Germany) with normalized laser power and filter settings making 1.5 and 0.5 μm thin optical sections.

### 2.3. Measurement of Protein Expression

**Figure 2:**
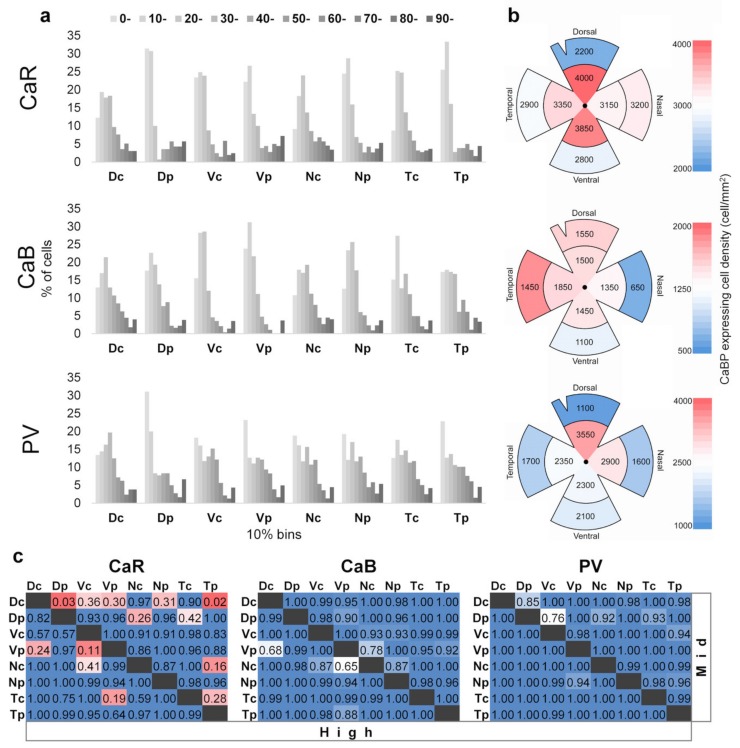
Topographical differences of CaBP expression in the mouse retina. (**a**) Expression intensity histograms. Individual bars show percentage of cells from the whole data subset and from the marked central or peripheral quadrant parts, those belonging to the 10% bin size of absolute expression intensity for each. A higher number of cells express lower levels (0%–30%) of CaR, CaB, and PV than at middle- (30%–60%) and high (60%–100%) levels in the dorsal (D), ventral (V), nasal (N), temporal (T), central (c), and peripheral (p) areas of the mouse retina (n = 5; 1727 RGCs, for variance see Figure S4). (**b**) The distribution density of CaBP expressing cells can be seen color coded. Higher densities are shown in red, average in white, and low values in blue, all three CaBPs are visualized using separate color scales to emphasize differences in the distribution within each CaBP group. In all cases a central-peripheral difference can be observed, showing higher densities towards the center. (**c**) Matrices show significance levels of a pairwise area-specific comparison in the expressional levels of CaBPs for medium (30%–60%) and high expression level (60%+) cells. Red outlines mark *p*-values that represent significant differences (*p* < 0.05). pink color represents close-to-significant *p*-values, while white and blue background suggests a no-to-small difference.

All measurements were performed using FIJI (NIH, USA, [22]). Firstly, a 100 × 100 µm square was defined in all Z-stacks for each retinal region, then z-merge was performed. The regions of interest (ROIs) for all retinal ganglion cells (RGCs) were selected based on the expression of NeuN (neuronal nuclei antibody) (Table 1; Figure 1), a neuronal marker which labels RGCs strongly and amacrine cells weakly in the GCL (Ganglion Cell Layer). To demonstrate this, we performed double staining using an RNA-binding protein with multiple splicing antibody (RBPMS, an RGC selective marker) and NeuN [23,24]. Mean gray value intensities and cell areas were measured. Intensity grey values were converted to relative percentage units by normalizing and rescaling values so that expression levels covered the 0%–100% range for all three proteins. These obtained relative percentage values were then sorted into 10% bins for all cells (Figure 2). Due to the nature of the z-merge, the measurable surface area is equal to the cell’s area at the widest cross section, henceforth referred to simply as area.

### 2.4. Statistical Analyses

One-way ANOVA analyses were performed using the Origin18 (Origin, version 2018b, OriginLab Corporation, Northampton, MA, USA). Normal distribution was previously confirmed through statistical analysis.

### 2.5. Clustering

All clustering was done using the Python SciKit-learn package [25] Gaussian Mixture Model (GMM), which uses an iterative process of expectation maximization (EM). This algorithm requires the number of clusters (K) to be provided, in order to determine the correct number of clusters represented in each dataset and the best covariance type (CT) of the model that fits the given dataset. The models were run with all available parameters (K = 1–20, CT = ‘diagonal’, ‘spherical’, ‘fitted’, ‘full’) and their respective bayesian information criterion (BIC) were calculated. Based on parsimony, the model with the lowest BIC was selected for that specific dataset.

### 2.6. Background Filtering

Three mean grey-values (GV) were obtained for each ROI based on expression levels of the three CaBPs. As ROIs were determined based on NeuN immunolabels, even non-expressing cells (negative for one or more CaBPs) were considered however their corresponding GVs were relatively low (equal to background staining). Therefore, we assumed that GV values of non-expressing cells fall in the lowest value GV cluster, and data corresponding to these clusters were handled as background in subsequent analyses. We used the gaussian mixture model (GMM) clustering method (described in more detail under the Clustering paragraph) to determine the background level, thus the non-expressing cells could be excluded from further analyses.

## 3. Results

In this study, the expression patterns of the three main CaBPs (PV, CaR, and CaB) were examined across the mouse RGC population in order to detect any protein-specific topographical distribution patterns. Both peripheral and central regions were used for measurements and further analyses from all four retinal quadrants (dorsal, ventral, temporal, and nasal) were performed for each examined retina (Figure 1a). All retinal regions are represented with a high number of samples (n > 5) in order to average out errors due to both individual differences between mice and also the potential methodological errors of immunolabeling. Please note that all steps of corresponding labels were highly standardized to avoid methodological artifacts. There are numerous cell types in the GCL of the retina, as shown by the DAPI co-labels in Figure 1c., thus we utilized the neuronal marker NeuN to determine neuronal and non-neuronal cells in our preparation. We also used the NeuN labels to define the region of interest (ROI) areas for further examination of CaBP expression levels. All NeuN-labeled RGC-somata, regardless of the CaBP content (or lack thereof) were considered in our analyses. Co-labels of NeuN, RBPMS, and GFP in the Thy1-GCaMP (THYmocyte differentiation antigen 1) mouse retinas show that RGCs were labeled with higher intensity (normalized grayscale values > 30%), while displaced amacrine cells (ACs) had a weak NeuN immunoreactivity (Figure 1d).

### 3.1. Expression of CaBPs in Mouse RGCs

For the first time, to our best knowledge, we show PV, CaR, and CaB expression in the same subset of neurons of the mouse retina, allowing for a direct comparison of the expression levels of the three CaBPs (see in Figure 1b). In general, the CaB labels of mouse RGCs were relatively weak compared to those of the CaR and PV labels. This observation did not result from poor antigen–antibody reaction or penetration problems, as horizontal cells in the deeper layers of the same retinal samples displayed remarkably strong labels (Figure S1). As our intention was not to compare expression levels of CaBPs to each other, but rather to standardize labeling intensities across samples, we normalized measured grayscale values (GVs) and provided them in a range of 0%–100%, where 100% was the maximum intensity level for each of the three markers. GVs were then displayed on histograms with a bin size of 10% (Figure 2).

In general, weak- and medium-labeled cells were the most numerous for all three CaBPs (Figure 2a; note that the graphs contain data from all ROIs, even those above background levels). Certainly, the most populous low-intensity bins contain a mixture of non-labeled and lightly labeled cells that thus provide the highest peaks of the histograms in most retinal areas. On the other hand, the brightest RGCs are likely composed of only a few (or only one) RGC subtypes as they form a considerably smaller population for all three CaBPs and their groups are represented by small but frequent well-separable peaks in the histograms. In addition to these general features, a few protein-specific patterns could also be discerned. One of them is the relatively high frequency of medium-intensity PV stained cells, whereas such intensities were less numerous for both CaR and CaB labels. As a result, the medium-intensity PV-positive RGCs formed additional peaks in the frequency diagrams in almost all retinal regions.

We performed an area-specific comparison for each CaBP to see if their expression levels are homogenous across the retina or display any regional specificity. The area-specific cell densities can be seen in Figure 2b, where a clear decrease in density towards the periphery can be observed. We found the lowest numbers and smallest centro-peripheral variation in the case of CaB. Regarding PV densities, a slight dorso-nasal preference can be found. The deviation from median expressions can be seen color-coded in Figure S5. The median value of CaR expression is higher to the dorsal side in the central retina, while PV-expression medians are higher in the ventral-central area. In the case of CaB and PV expression medians, variance could be detected between different topological regions (Figure S5).

We utilized the data from only the medium- and strongly labeled cells for this analysis. We found that the expression levels of CaB and PV are rather homogenous, whereas the expression of CaR varies somewhat (Figure 2c). More specifically, there is an observable eccentric specific pattern difference in the CaR expression in the dorsal, nasal, and temporal regions (Figure 2a,b). Individual variances in mean SDs are as follows: CaR 6.5, CaB 3.18, and PV 9.16 (see Figure S4. for details).

In order to demonstrate these expressional differences more precisely, we created three categories based on the relative intensity levels for each CaBP, namely ‘high’, ‘medium’, and ‘low’ (high = 60–100th percentile, medium = 30–60th percentile, low = 0–30th percentile). Pairwise comparison and a corresponding statistical analysis (n = 8; one-way ANOVA) was then carried out to determine any CaBP expressional difference between retinal regions (Figure 2c; numbers in the table represent the significance level of the difference, >0.5 shown in blue, <0.05 shown in red).

We found that expression levels of PV and CaB were rather homogenous across retinal regions, confirming our qualitative observations. However, we found a higher number of mid-intensity CaR expressing cells in Dc (Dorsal-central) areas when they were compared to those of Dp (Dorsal-peripheral) and Tp (Temporal-peripheral retinal areas. In these two comparisons, the differences were statistically significant (Dc/Dp: *p* = 0.03; Dc/Tp: *p* = 0.02, One-way ANOVA). Moreover, indicative differences were also found between the Vc/Vp (Ventral-central/-peripheral) regions in the high-intensity (GV > 60%) subset (Figure 2a,c) of CaR expressing cells. The Dc area contains a higher number of medium-labeled CaR+ cells as well, compared to other regions (Dp—28%; Vc—20%, Vp—17%, Np—21%, Tp—25%). In addition to Dc, the Nc (Nasal-central) area also maintains a somewhat higher number of medium-labeled CaR expressing RGCs than the Dp (20%) and Tp (17%) areas. The Vp and Nc areas also displayed a somewhat higher number of highly stained CaR+ RGCs when compared to numbers in the Dc (5%), Vc (8%), and Tc (7%) locations (Figure 2a,c). However, the observed differences in these latter three comparisons were only indicative according to our statistical analysis. Altogether, it appears that the central retinal areas in the dorsal and nasal quadrants maintain a higher number of CaR expressing cells mostly among the medium-labeled RGCs. However, all things considered, the measured protein expression levels indicate no topographical differences in the distribution of CaB and PV in RGCs, suggesting that their importance and function is also uniform throughout the retina.

### 3.2. The Soma Size Distribution of CaBP Expressing RGCs

Based on the above first set of experiments, we suspected that low-expressing cells in our dataset blend in with the background staining of the tissue. Therefore, prior to further analysis, we cleaned up our dataset with a background filtering process (see Section Methods; Figure S2). First, we performed a cluster analysis based on CaBP-labeling intensities of RGCs. We assumed that labeling intensities of non-expressing cells (background staining) fall in the lowest GV cluster, therefore data corresponding to these clusters were merged with the background and RGCs comprising these clusters were handled as non-expressing cells in the subsequent analysis. Next, the relative frequencies of CaBP expressing RGCs were determined for each examined area. Approximately 25% of all RGCs expressed CaB, more than half of them were positive for CaR and 25%–53% of cells were labeled with the anti-PV serum. The greatest centro-peripheral difference was observed for PV+ RGCs in the dorsal-retinal quadrant where only 25% and 53% of RGCs expressed PV in the peripheral and central areas, respectively (Table 2).

**Figure 3:**
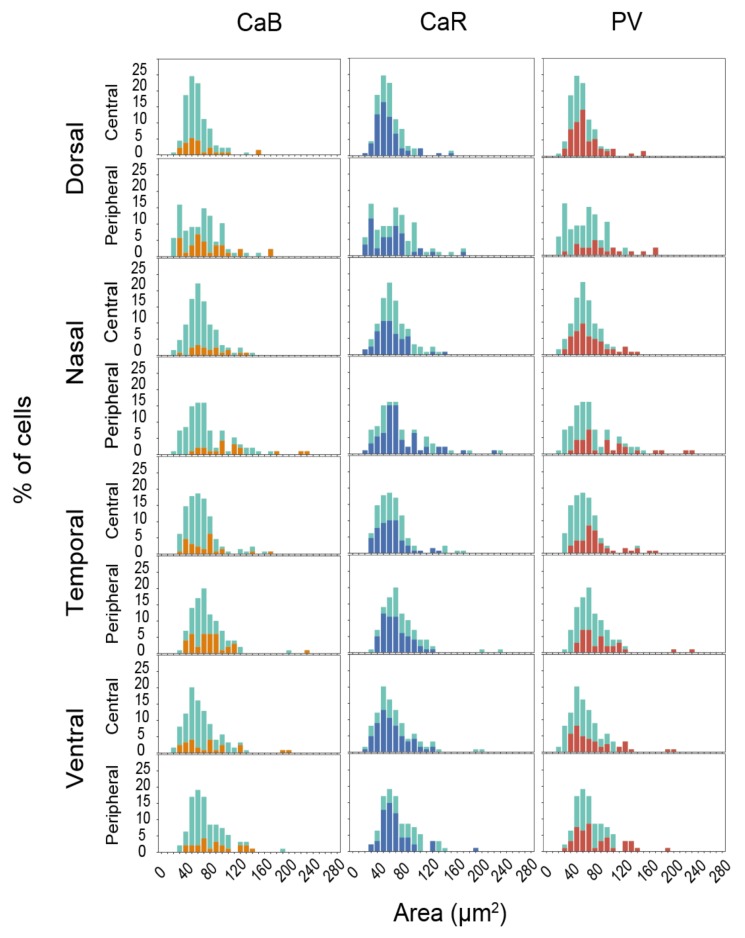
Soma size distribution histograms of CaBP expressing RGCs. The cell size distribution of all RGCs (light blue) and CaBP expressing RGCs (CaB: orange, CaR: blue, PV: red). The bin widths are set at 10 µm^2^. Note, that only data from quadruple labeling experiments (CaR, CaB, PV, NeuN) were used for this analysis, thus light blue histograms (all cells) are the same across all rows present.

In the second set of analyses, we measured the area of somata, which we expressed in µm^2^ for all RGCs, and then compared the distribution histograms of CaBP expressing and non-expressing cells. This analysis showed that somatic area histograms of CaBP expressing RGC populations fell into a range as wide as those generated for all RGCs. Only slight differences could be detected in case of the CaB and PV expressing RGCs that tend to fall in the right halves of the histograms (larger cells) in certain areas (Figure 3; CaB—Nc, Np; PV—Dp, Np, Tp, Tc, and Vc). However, these observed differences proved statistically insignificant and it appears that all three CaBPs can be expressed by RGCs with any soma size. This finding further indicates that the three populations of CaBP expressing RGCs are heterogeneous and contain several functional RGC subtypes.

### 3.3. Clustering RGCs Based on CaBP Expression and Soma Sizes

**Figure 4:**
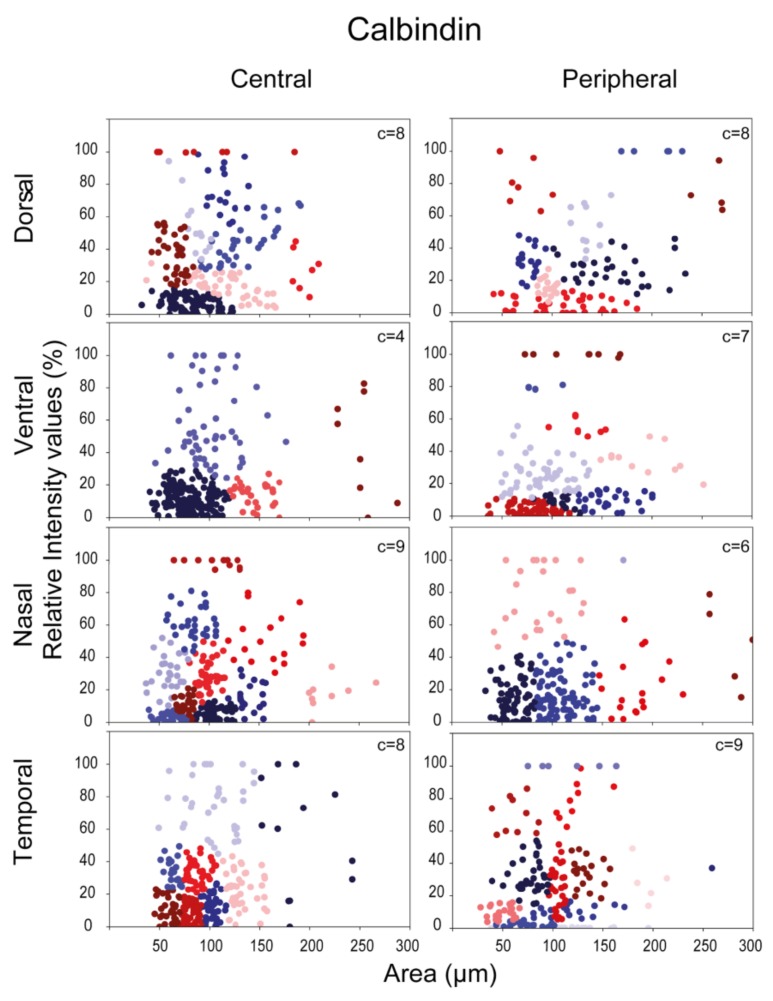
Cluster analysis of CaB expressing RGCs. Two features (RGC soma size and CaB expression level) were utilized to perform the clustering in both central (left) and peripheral (right) retinal areas in each quadrant. Dots indicate individual cells from n = 8 retinas. The clusters of RGCs are color-coded (note that colors do not necessarily represent the same cluster in separate panels). The number of clusters are shown in the top right corner of each graph.

**Figure 5:**
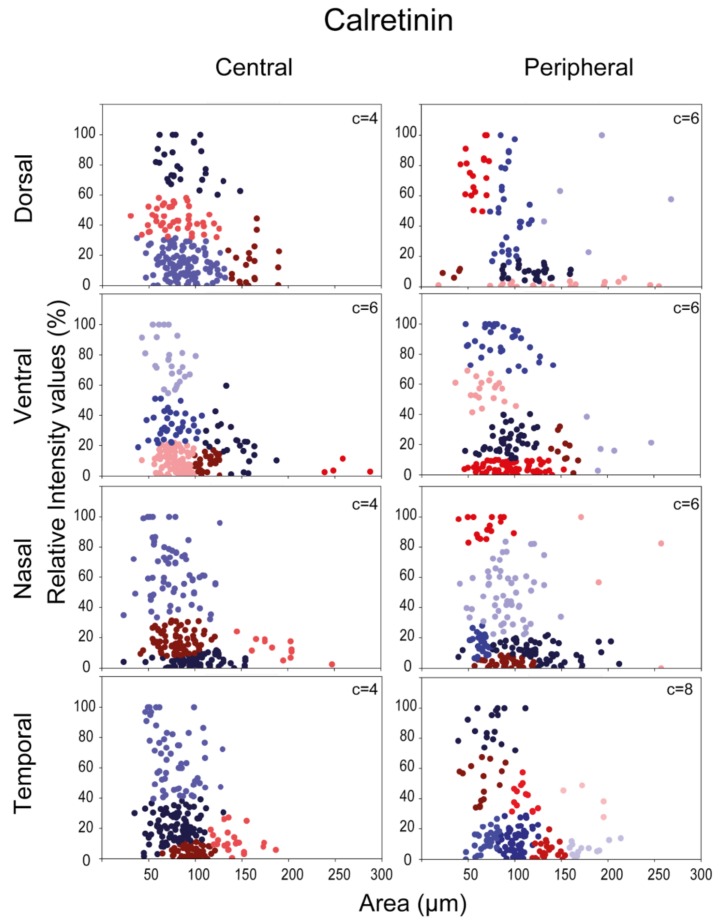
Cluster analysis of CaR expressing RGCs. RGC soma size and CaR expression level were utilized to perform the clustering in both central (left) and peripheral (right) retinal areas separately for each quadrant. Dots indicate individual cells from n = 5 retinas. The clusters of RGCs are color-coded (note that colors do not necessarily represent the same cluster in separate panels). The number of clusters is shown in the top right corner of each graph.

**Figure 6:**
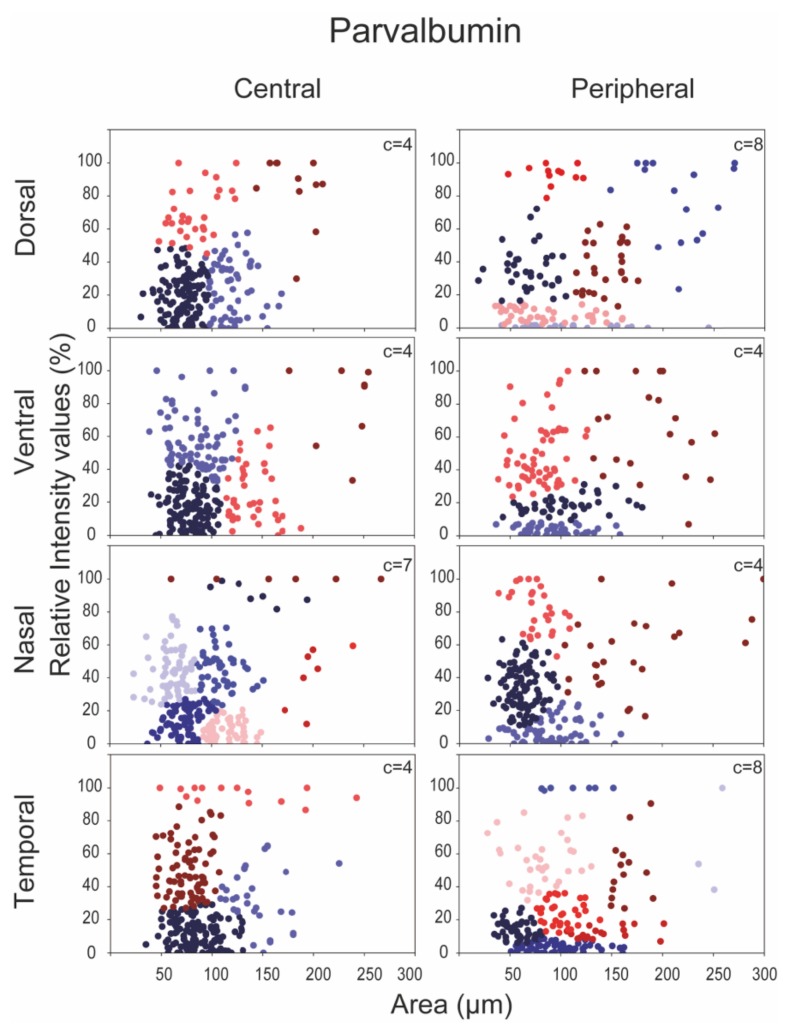
Cluster analysis of PV expressing RGCs. Two features, RGC soma size and PV expression level, were utilized to perform the clustering in both central (left) and peripheral (right) retinal areas separately for each quadrant. Dots indicate individual cells from n = 6 retinas. The clusters of RGCs are color-coded (note that colors do not necessarily represent the same cluster in separate panels). The number of clusters is shown in the top right corner of each graph.

In a subsequent analysis we plotted expression levels of CaBP-positive RGCs as a function of somatic areas; central and peripheral areas were examined separately in order to find any potential differences. In fact, as shown in the scatter plots below (Figure 4, Figure 5 and Figure 6.), we found a differential expression pattern when central and peripheral regions were compared. We defined subgroups of RGCs in each CaBP-stained population based on the same two parameters—the labeling intensity, and the soma size—with cluster analysis (see Materials & Methods), and presented the clusters in scatterplots of Figure 4, Figure 5 and Figure 6. (For direct central-peripheral comparison see Figure S3.)

This analysis resulted in four to nine RGC subpopulations for all three CaBPs (CaB: four to nine; CaR: four to eight; PV: four to eight) that varied somewhat depending on the retinal location. These observed location-specific inhomogeneities obtained for a particular protein strongly suggest that the clusters are not equivalent to morphological/functional RGC subtypes. However, the analysis further supports our previous results and also prior experimental work [11,26] showing that the three examined CaBPs are expressed by a heterogeneous group of mouse RGCs. While the number of clusters may not be equivalent to CaBP expressing cell types, these numbers can still provide a range to compare to morphological clusters. Moreover, in many cases, scatterplots display cell groups (clusters) that can easily be pointed out simply by eyeballing the presented data. These latter, well-separable clusters likely define one or two RGC subpopulations that can easily be differentiated from the rest of the cell groups in the immunohistochemistry (IHC)-stained specimens as well. In addition, it is also evident from this analysis that peripheral scatterplots are not simply right-shifted versions of their central counterparts. The existence of such a shift would only be expected if there was a significant centro-peripheral eccentricity-dependent soma-size change of cells in the same functional RGC population. Interestingly, Tp areas contained the greatest number of clusters for all three CaBPs, suggesting either a better separation of RGC subtypes here or a higher internal variability of CaBP expression in one particular RGC type. Besides the Tp, only the nasal-retinal quadrant showed high cluster numbers in the case of CaB and PV (Figure 4, Figure 5 and Figure 6). Besides these general occurrences, we made the following observations: the CaB+-Vc area, for example, has a population of cells characterized by small somata and high CaB expression levels. These cells seem to be only present in small numbers on the periphery (only 10 cells). We also observed a higher number of RGCs with large somata in Dp, Vc, and Np areas whose CaB expression levels varied considerably. Interestingly there were some CaR-labeled cells with large somata and high CaR expression in the Dp and Np areas, whereas other areas showed only low CaR expression among the population of large somatic cells (Figure 5). Histograms of PV+ cells displayed a greater variability in size on peripheral regions than in central areas, but cells with large PV+ somata could not be separated into any further groups based on labeling intensities alone. In summary, sole expressional data derived from CaB, CaR, and PV labels are not sufficient for defining functional RGC cell types in mouse retinas. To sum it up, it is difficult to draw any conclusion from the observed centro-peripheral changes without morphological identification of the involved RGC subtypes—our data show a topological divergence between expression clusters of RGCs.

### 3.4. Expression of Multiple CaBPs

**Figure 7:**
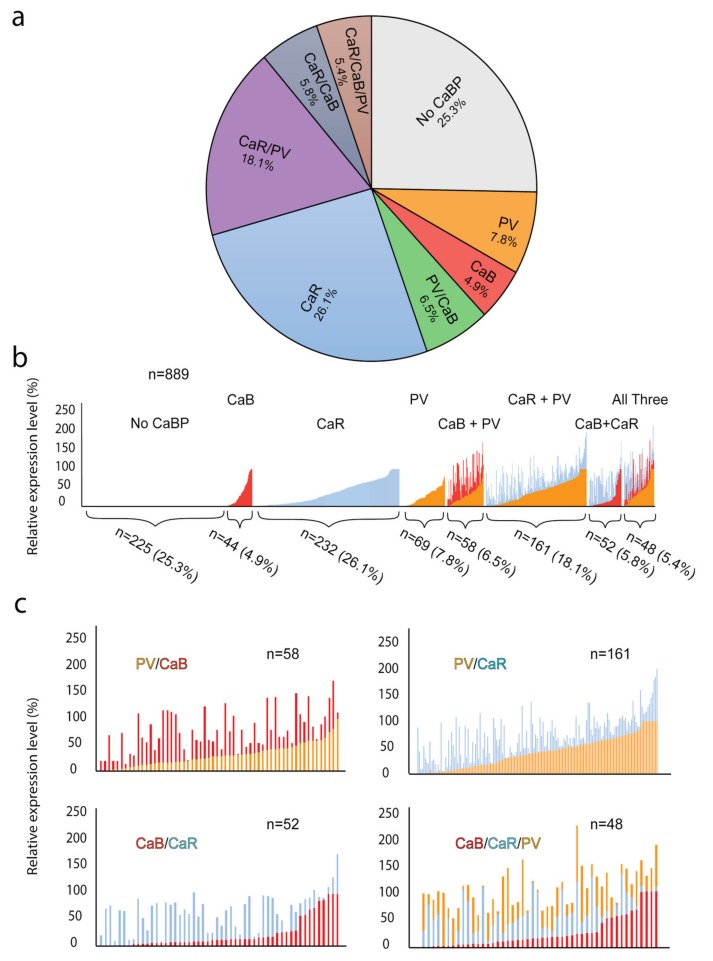
Comparison of multiple CaBP expressions in triple-labeled cells. Panel (**a**) showing a pie chart representing the ratios of the RGCs on the basis of CaBP expression (none, singularly, double and triple expressing) for the RGCs in our sample. Panel (**b**) is a stacked bar-chart, where the *y*-axis is the relative expression level of the three studied CaBP (CaB = red, CaR = blue, PV = orange). A closer view of the double and triple expressing population can be seen in the panel labeling individual cellular expression of CaBPs (**c**).

Our next goal was to examine if the expression of the three CaBPs overlap, forming RGC populations where individual cells co-express two or more CaBPs simultaneously. To this end, we performed quadruple-labeling (NeuN, CaR, CaB, and PV) experiments which allowed us to measure expression levels of all three CaBPs in the same RGC population (Figure 7). Following the application of the background correction algorithm (see Section Materials and Methods for details), expression levels of CaBPs were determined for a set of labeled RGCs (n = 889; taken from all regions of n = 3 retinas). We found that about a quarter (25.3%) of our RGCs showed no CaBP label at all, whereas about one third of the cells were labeled by only one of the sera. Among these latter single-labeled cells, CaR+ RGCs were the most numerous (26.1%), whereas sole CaB or PV was expressed by smaller groups only (4.9% and 7.8%, respectively). Besides RGC populations expressing only one CaBP, we also found a sizeable population of CaR+/PV+ cells (18.1%), as well as smaller groups of CaB+/PV+ (6.5%) and CaR+/CaB+ (5.8%) dually labeled cells. Finally, a small group of RGCs (5.4%) showed triple labeling in our sample (Figure 7a). Cells were also sorted based on their CaBP expression levels in order to examine any possible correlation between intracellular protein levels (Figure 7b,c). Based on this comparison, we made the following observations: (i) a small population of the CaR+/PV+ RGCs expressed both proteins in relatively high levels; (ii) there is a seemingly inverse relation of the CaR and CaB levels in the dually labeled CaB+/CaR+ RGC population; and (iii) the inverse relation of CaB and CaR levels can be detected in cells of the triple-labeled CaB+/CaR+/PV+ population as well (Figure 7b,c). According to this latter observation, cells with relatively bright CaR labels showed a faint signal for CaB, whereas cells with relatively good CaB staining presented a weaker CaR signal. At the same time, many RGCs displayed intermediate label intensities for both CaR and CaB.

### 3.5. Correlation of PV Genetic Markers to the PV Antibody Labeling

**Figure 8:**
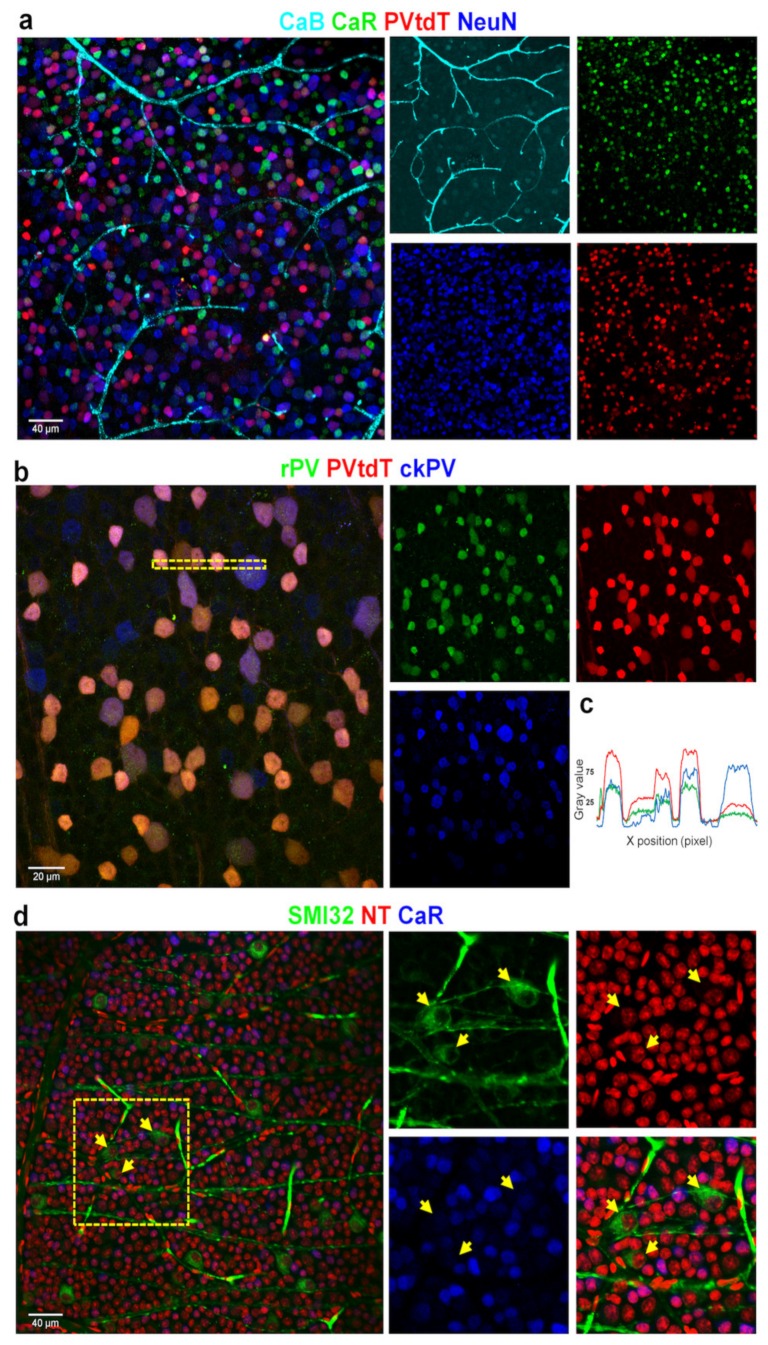
Correlating multiple labels with CaBPs can help reveal individual differences between antibodies and GC identity. Panel (**a**) shows the parvalbumin-tdTomato (PV-tdT) GMO mouse retina with CaB, CaR, and NeuN labeling (composite image on the left and split into four channels on the right—all colors correspond to the label above). Panel (**b**) displays the comparison between PV-tdT, rabbit-PV (rPV), and chicken-PV (ckPV) antibody-labeling performed on the same retina. (**c**) A histogram profile, acquired from the dashed area in b shows a variance in normalized relative intensities of the PV-tdT (GMO, red) and r- (green), ckPV (blue) labels. (**d**) ON and OFF GCs (ganglion cells) are brightly labeled with SMI32 (arrows) but not by CaR. CaR, on the other hand, labels smaller GCs not labeled by SMI32. Neurons and vascular cells are all labeled with the fluoro-Nissl stain Neurotrace-640/660 (NT).

One may question how descriptions of CaBP labels in different studies utilizing different antisera can be matched. In addition, GMO (Genetically Modified) animals with markers under the control of CaBP promoters may show discrepancies when compared to IHC samples. To examine this latter issue, we utilized retinas of the parvalbumin-tdTomato (PV-tdT) mouse line (see Section Materials and Methods) and performed a CaB/CaR/NeuN quadruple-labeling experiment where the PV-IHC label was substituted with the PV-tdT staining. We found that the labeling patterns of all three markers in this experiment (Figure 8a) were rather similar to those of quadruple-label IHCs (see also Figure 1a).

As pointed out above, different antisera may result in dissimilar labeling patterns. To examine this issue, we performed a dual IHC experiment in our PV-tdT mice by using two a-PV sera with different hosts (rabbit—r, chicken—ck). Interestingly, label intensities of PV+ RGCs appeared different when comparing the slides stained with the two PV-specific antibodies. The most obvious difference was the low-intensity labeling of the large-cell-bodied RGCs with the rabbit hosted serum, whereas the same cells displayed strong labels with the chicken a-PV antibody (Figure 8b). However, we observed no single-labeled PV+ RGCs in our sample, which in turn confirms the specificity of the primary sera raised in two different host species. 

## 4. Discussion

### 4.1. Comparison with Existing Descriptions

RGCs in the mammalian retina have been described previously as heterogeneous populations for all three examined CaBPs (reviewed in [1]). Therefore, none of them can be utilized effectively as a subtype-specific RGC marker in retinal morphology studies. However, CaBP labels have proved useful for labeling retinal layers [27] or, combined with other markers, served as a methodological approach to selectively examine a subset of the RGC population. One such combined approach was presented in a series of studies by the Jeon laboratory. They carried out intracellular dye/tracer injections into RGCs that were immunohistochemically labeled to CaR [11,28], CaB [12], or PV [26]. In these studies, CaBP expressing RGC morphologies were described in detail, identified morphologically, and sorted into six to eight RGC classes. Morphological descriptions of PV expressing RGCs have also been performed by the dye injection of RGCs stained in GMO mice where GFP fluorescence of cells was PV-promoter-dependent [9]. Besides a few discrepancies (e.g., existence of OFF alpha cells in the GMO but not in the IHC study among PV expressing cells), these two studies were largely consistent in the number of defined PV expressing RGC subtypes, as both differentiated eight different groups in the examined RGC population. We tested our own PV-Cre x tdTomato line (see Methods) and found that PV expressing RGCs in the GMO mice display a 100% match with our a-PV IHC experiments, even though the labeling intensity could differ between certain cell populations (Figure 8). This latter result further confirms the high degree overlap between genetic and IHC markers in the case of PV.

Similar to the above morphological descriptions, our cluster analysis also determined eight RGC groups based on soma size and PV expression levels in the Dp and Tp retinal areas, whereas other areas had seven or four PV immunoreactive RGC clusters. At this point, it is uncertain whether the observed PV+ clusters (at least in the Dp and Tp regions) are in a one-to-one relation with the above morphological analyses. Soma sizes often show a strong correlation to the diameters of corresponding dendritic fields, therefore it is likely that PV-IR RGC subtypes with smaller dendritic fields in the morphological analyses correspond to cells of small somata of this study, and cells with larger cell bodies maintain larger dendritic arbors as well. In fact, morphological analyses differentiated to three large-field (PV1, PV5, PV6 [9]; PV7, PV8 [26]) and five to six small/medium-sized (PV2, PV3, PV4, PV7 [9]; PV3, PV4, PV5, PV6 [27]) PV expressing RGC populations. Moreover, these RGC populations corresponded well to our cluster analyses in the Dp/Tp areas, where we found one to two clusters of RGCs with large somata and more than five clusters with small/medium-sized cell bodies. This putative correlation is weaker in the rest of the retina (Dc, Nc, Tc, Vc, Np, and Vp areas) with <8 identified clusters.

Lee and colleagues have identified 10 morphologically distinct CaR expressing RGCs in the mouse retina by using the above-mentioned combined IHC/dye injection method [11,26]. The identified cells were mostly RGCs with small- or medium-field dendritic arbors. Our results were consistent with this description as CaR-IR RGCs in our sample fell mostly in the clusters with small to medium soma sizes (<200 μm^2^ area surface; assuming that soma and dendritic arbor diameters are largely correlated; see Figure 4, Figure 5 and Figure 6). According to the morphological analysis by Lee and colleagues, two additional RGC populations (CR10 ON and CR10 OFF) had large dendritic arbors and they showed morphological characteristics similar to ON and OFF alpha RGCs in the mouse retina [3,29]. This is consistent with studies in which a GMO mouse line was utilized, where the presence of the CaR promoter in the animal induces GFP expression in OFF alpha RGCs [30]. Our dataset also contained a small population of RGCs with relatively larger somata (>120 μm^2^ surface area) and low CaR expression levels. This population was present in all retinal areas and was less populous than other clusters. The relatively low abundance of these cells points to the conclusion that they are, in fact, alpha cells that are less numerous than most other RGC subtypes in the mammalian retina [31] The SMI32 antiserum has been shown to selectively label ON and OFF alpha ganglion cells in the mouse retina [32,33,34]. In fact, our SMI32/CaR/NeuN triple-labeling experiments (Figure 8) showed a weak or no expression of CaR in a subset of SMI32-positive RGCs (Figure 8c), whose visible soma-dendritic morphology resembled the ON alpha RGCs [3,29,35]. Therefore, we conclude that one of our large-cell-bodied CaR expressing populations was likely the ON alpha RGCs. In this scenario, the cluster of ON alpha RGCs was distinguishable in all retinal areas, forming a weakly labeled CaR+ population with relatively large somata. This result could prove useful in identifying the population of ON alpha RGCs in the mouse retina.

CaB has been shown to be expressed by 10 RGC populations according to the only relevant morphological analysis covering this issue [12]. This, again, outnumbers the clusters detected in our analysis (4–9 in various retinal regions). It is clear that our cluster analysis could not separate efficiently the various CaB expressing RGC subtypes that were merely based on the CaB expression levels and soma area measurements. Based on the results presented by Gu and colleagues, A2 RGCs (equivalent to alpha RGCs) were the only large soma/large dendritic arbor cell type that displayed CaB positivity. This suggests that the cells with the largest CaB+ somata in our analysis may, in fact, correspond to these alpha RGCs. If so, then ON alpha cells are among the weakest CaR expressing RGCs in the mouse retina but at the same time, they are relatively well-labeled with the CaB serum (note that the a-CaB serum produced an overall weak label for all mouse RGCs).

Based on previous IHC/cell injection studies [11,12,26], one may conclude that besides the few RGC types that were not CaBP+ or shown expressing only one of the three CaBPs, most were dual- or triple-stained [1]. However, the authors also stated that only a certain ratio of a single RGC subtype expressed a given CaBP (e.g., 23.83% of ON-OFF directionally selective RGCs were CaB+, 31.58% CaR+, and only 11.59% PV+). This suggests that rather than being multiple-labeled, a certain RGC subtype may contain subpopulations that express either one of the examined proteins. Our study here showed data for the existence of RGC populations with dual and triple CaBP labels and thus provides a direct answer to this question. Our findings do not rule out the possibility of the existence of subpopulations in a certain RGC subtype, but definitely show that dual- and triple-labeled RGCs exist in the mouse retina in a relatively high number. These data, therefore, indicate a diversity of CaBP functions and a shared labor and/or subcellular compartmentalization of these proteins that potentially necessitates the expression of multiple CaBPs in a single cell.

One interesting finding was an inverse relationship of CaB and CaR levels in both the CaB+/CaR+ and the CaB+/CaR+/PV+ RGC populations (Figure 7), suggesting a complementary intracellular function of these two proteins in mouse retinal RGCs. In fact, the results of two separate studies from the Jeon laboratory [11,12] attested that the same ten mouse retinal RGC subtypes were found expressing CaR and CaB as well. They also found that the highest number of CaR expressing RGCs was in the central retinal regions whereas CaB expressing cells were more numerous in the mid-peripheral retinal areas. Thus, each protein had its maximum in locations where the other protein displayed a minimum. Although CaB/CaR dual staining was not performed in these studies to test this possibility directly, these results strongly indicate that the same RGC population (cells of the ten subtypes) express both CaB and CaR, and the CaB/CaR expression ratio varies as we move from the center of the retina towards the periphery, meaning that the CaB/CaR ratio increases with eccentricity. On the other hand, the expression of PV does not seem to show a similar correlation to either of the other two proteins. However, no examples of the same ten RGC subtypes presenting standalone CaR+ labeling in the center and CaB+ afar have been presented as of now. Nevertheless, our observation combined with previous studies strongly suggests the presence of mutual substitution mechanisms in RGCs. According to this hypothesis, the weak CaR expression of RGCs is often combined with strong CaB expression and vice versa. If this hypothesis holds, alpha RGCs may comprise one of the example groups. The close structural homogeneity between the two proteins [5] could also provide a basis for the assumed complementary function, allowing for a more sophisticated tuning of the region or subtype specificity. Moreover, the combined data further indicate that there is a slight variation in the intracellular performance (or role) of the two CaBPs, by which one is favored in more central regions (CaR) whereas the other protein is more prominently expressed in the peripheral counterparts (CaB) of the same RGC subtypes. This second hypothesis needs to be investigated in the future as well.

### 4.2. The Topology of CaBP Expressing RGCs in the Mouse Retina

One of the main goals of this study was to compare the expression levels of the three main CaBPs in an extended retinal surface and detect any potential area-specific distributional variety in terms of either the expression levels or the frequency of CaBP expressing RGCs. We observed that in general, the CaB expression level was considerably lower across the whole RGC population than levels of either CaR or PV. The lower intensity labeling of CaB was not the result of poor IHC staining or penetration problems because the same serum provided a bright label in horizontal cells of the same retinal samples (even though they are located in the middle of the tissue). Therefore, we conclude that the weak CaB staining in RGCs is due to the low levels of intracellular CaB protein molecules. In contrast, both the anti-CaR and anti-PV sera provided bright labels for at least one RGC population. Overall, we found that the a-CaB labels resulted in the weakest RGC staining and the least number of stained cells out of the three studied CaBPs. This was consistent with previous descriptions [12].

Expression and lack of expression for a certain RGC subtype have been shown for all three CaBPs in the previous morphological studies. We show similar data for regional CaBP expressing cell densities (Figure 2b)—our findings are consistent with the results obtained by the Jeon lab, published previously. The expressional tendencies are identical, with the caveat that their results have a higher resolution. The cell number ranges are slightly different as compared to them we generally measured lower numbers for CaR expressing cells and equal or higher numbers for PV and CaB expressing cells. These differences however are most likely explained in the case of CaR by the fact that we excluded amacrine cells from our experiment (using the NeuN labelling). In the case of PV and CaB we probably found higher numbers, because we included cells that showed low expression levels as well [11,12,26]. These findings may explain the inconsistency of cluster formation of CaR-, CaB-, and PV-labeled RGCs in the eight retinal areas of this study (Figure 4, Figure 5 and Figure 6.).

Contrary to the findings that RGCs belonging even to the same subtype can display diverse staining for a certain CaBP label, we found that the overall distribution of CaBPs was relatively homologous in the quadrant/eccentricity-specific comparison. This finding is in apparent conflict with previous results showing the above-mentioned centro-peripheral density decrease in the numbers of CaR+ and PV+ RGCs [11] and a local mid-peripheral maximum of CaB+ RGCs [11]. In our study, however, it was not the absolute cell counts but rather the relative density of RGCs with low, medium, and high label intensities that were compared (Figure 2c). As a result, our region-specific intensity measurements are not necessarily in conflict with previous descriptions. According to our findings, only CaR expression levels displayed some area-specific heterogeneity, significantly favoring medium-labeled cells in Dc areas vs. Dp and Tp regions (Figure 2c). In addition, Nc areas also showed a somewhat higher number of medium-labeled CaR+ somata when a comparison was performed with the Dp and Tp regions. Therefore, both Dc and Nc regions showed a higher frequency of medium labeled CaR+ RGCs when compared to most peripheral regions (Tp, Vp, and Np). We believe that this finding is related to the centro-peripheral drop of the CaR+ cell count described by Lee and colleagues [11]. Contrary to this, a higher number of brightly labeled CaR+ cells were observed in the Vp region when the comparison was performed with central regions (Dc, Vc, and Tc). This seemingly contradicts the above-mentioned eccentricity-driven peripheral drop of CaR+ cells. However, considering that brightly labeled CaR+ RGCs only provide a small fraction of the total number of CaR+ RGCs, the centro-peripheral increase of their number (which is the exact opposite of those of the medium-labeled cells) likely does not affect the overall changes of CaR+ RGC numbers. In contrast to the area-specific expression level differences of CaR+ RGCs, we observed no evident expressional variations for PV or CaB levels. This fact might not entirely be surprising in the case of CaB where the highest cell counts can be found in the mid-peripheral retinal regions [12] that contributed to both central and peripheral retinal regions in our comparison, thus impeding us to find similar area-specific variation in CaB expression. The homogenous expression of PV in mouse retinal RGCs is somewhat surprising in light of the described centro-peripheral drop of PV+ RGC numbers [26], but again, the previous morphological analyses were performed based on cell counts while this study, in turn, measured CaBP expression levels and therefore the two sets of values cannot be directly compared.

Until recently, the mouse retina has been considered relatively uniform regarding RGC distribution. However, the work published by El-Danaf et al. shows that RGC subtype density can be subject to localization [36]. W3 cells in the mouse (categorized as B2 by Sun et al. [33] and CR1, CB1 by Jeon and colleagues [11,12]) display a certain level of eccentricity in their distribution which can also be observed in CaBP expression (see Figure 2 for easy visualization). However, considering that our study prioritized labeling intensities over density, it would be unwise to draw any direct conclusions. ON-OFF direction-selective RGCs (D2 by Sun et al. [37] and CR6 and CB3 by Jeon and colleagues [11,12]) displayed higher densities towards the ventral regions [36] but we found no clear ventral preference for the examined CaBPs in this study, further reinforcing that CaBP distribution is not in direct correlation with subtype-specific RGC localization.

Altogether, based on the combination of the above results and also the findings described in previous reports, we conclude that none of the examined CaBPs are expressed in a subtype-specific manner. There are a number of RGC subtypes that can express one or more CaBPs and individual RGCs of a certain subtype may appear very different in terms of expression levels, however, our results show that clusters of CaBP expressing cells could be defined in future multiple label studies.

## Figures and Tables

**Table 1:** Antibodies.

Primary Antibodies				Secondary Antibodies, Dyes			
Name	Dilution	Source	Code	Name	Dilution	Source	Code
rb-Calretinin	1:2000	Invitrogen	180211	anti-rb-Alexa647	1:500	Invitrogen	A21245
ms-Calbindin	1:1000	SySy	214 011	anti-ms-Alexa488	1:1000	Invitrogen	A11017
ck-Parvalbumin	1:250	SySy	195 006	anti-ms-Cy3	1:500	Jackson	115-165003
rb-Parvalbumin	1:500	Thermo	PA1-933	anti-gp-DyLight405	1:500	Jackson	706-475-148
gp-NeuN/Fox 3	1:1000	SySy	266 004	anti-ck-Alexa568	1:500	AbCam	ab175477
ms-SMI32	1:1500	Calbiochem	NE1023	anti-rb-DyLight405	1:500	Jackson	711-475-152
rb-RBPMS	1:1000	Abcam	ab152101	DAPI	1:20,000	Sigma	D9542
				NeuroTrace 640/660	1:1000	Thermo	N21483

**Table 2:** Relative frequency of given protein-expressing cells (given as a percentage of all RGCs in the corresponding retinal region).

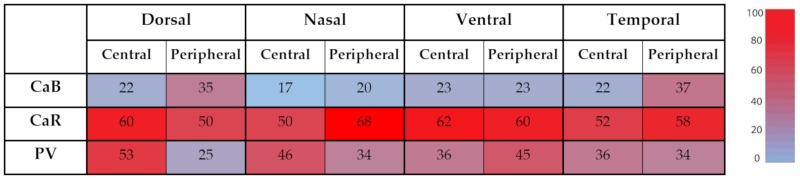

## Supplementary Materials

The following are available online at https://www.mdpi.com/2073-4409/9/4/792/s1; Figure S1: Weak CaB expression in mouse RGCs are not the result of antibody specificity or penetration problems. The image panels show CaB-stained RGCs and horizontal cells (HC) in the same frame of the mouse retina focusing on the ganglion cell layer (a) and the depth of the distal INL/OPL area (b). Even though superficially locating RGCs show weak CaB staining, deep-layer HCs are heavily labeled with the same serum contrary to the fact that these cells are in the middle of the tissue (prone to penetration problems). Arrows and asterisks label the same cells on the side view. Clearly, the weak CaB staining in RGCs is not a methodological artifact. Figure S2: Plots show two examples of scatterplots in which cells were ranked according to their CaBP expression levels. Such plots were utilized to define ‘first-cluster background labels’ that allowed us to further minimize our expressional analyses. Figure S3: Scatterplots display expression level/soma size relations for RGCs in central and peripheral retinal areas in each quadrant (central: blue, peripheral: red). The same scatterplots were also utilized to define RGC clusters (see Figure 4, Figure 5 and Figure 6). Figure S4: Variance between expression bins between individual retinal samples measured on the same dataset used in Figure 2. n = 5 (nr. of mice); 1727 RGCs, 10% expression bins for CaR, CaB, PV. Figure S5: The deviation from median expression color coded. Red represents cell numbers with higher expression, lower numbers with blue and white closest to the median. All cases, except the CaR and CaB ventral quadrant, show a stronger expression in the central part of each quadrant. Variance in CaR expression is observable between the center and periphery. In the case of CaB and PV, some variance could be detected which is restricted only to the difference from the median.
