# Supplementary material for: Spatial Expression Pattern of the Major Ca2+-Buffer Proteins in Mouse Retinal Ganglion Cells
Source: Cells. 2020 Mar 25;9(4):792. doi: 10.3390/cells9040792 (PMC7226302; doi:10.3390/cells9040792)
Supplement: Supplementary file 1 [file cells-09-00792-s001.pdf]

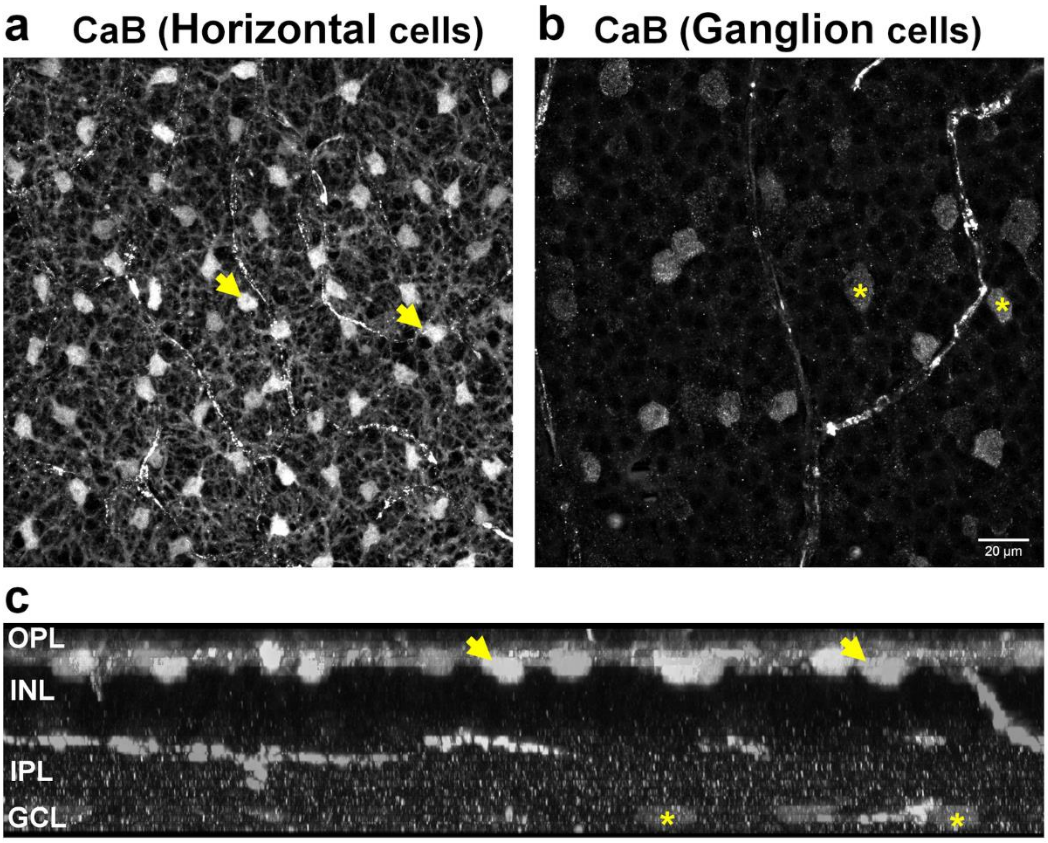

Figure S1: Weak CaB expression in mouse RGCs are not the result of antibody specificity or penetration problems. The image panels show CaB stained RGCs and horizontal cells (HC) in the same frame of the mouse retina focusing on the ganglion cell layer (a) and the depth of the distal INL/OPL area (b). Even though superficially locating RGCs show weak CaB staining, deep-layer HCs are heavily labeled with the same serum contrary to the fact that these cells are in the middle of the tissue (prone to penetration problems). Arrows and asterisks label the same cells on the side-view. Clearly, the weak CaB staining in RGCs is not a methodological artifact.

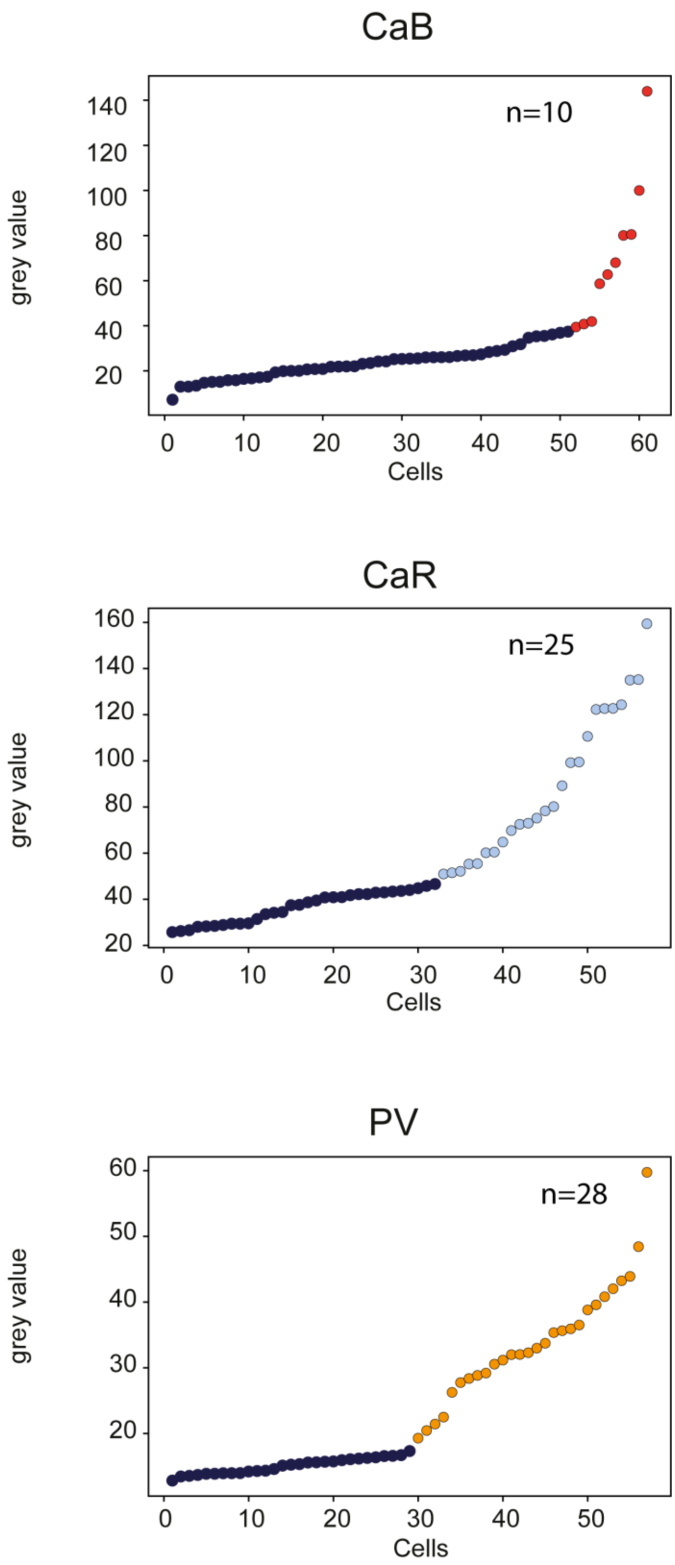

Figure S2: Plots show two examples of scatterplots in which cells were ranked according to their CaBP expression levels. Such plots were utilized to define 'first-cluster background labels' that allowed us to further minimize our expressional analyses.

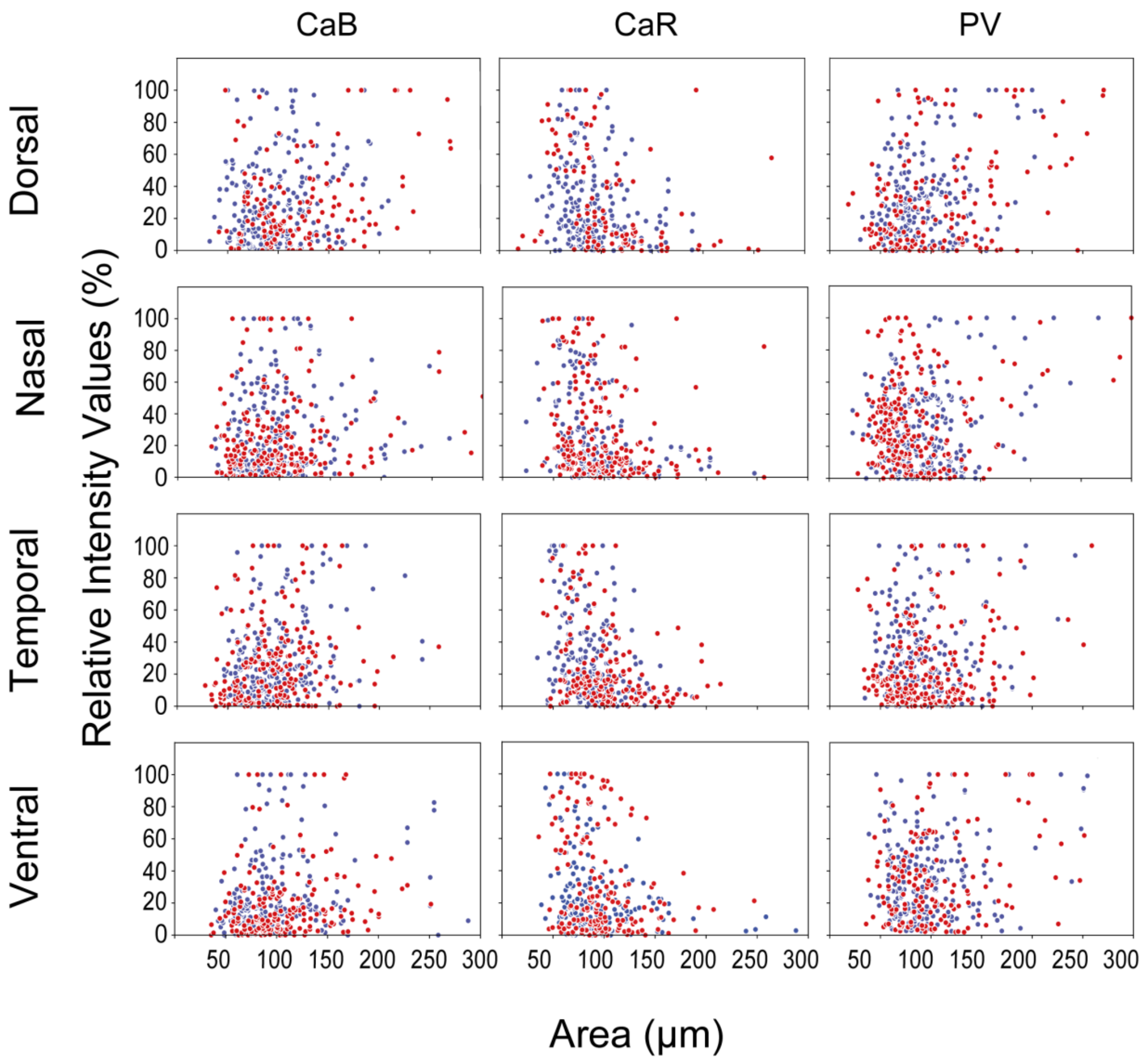

Figure S3: Scatterplots display expression level/soma size relations for RGCs in central and peripheral retinal areas in each quadrant (central: blue, peripheral: red). The same scatterplots were also utilized to define RGC clusters (see Figures 4, 5 and 6).

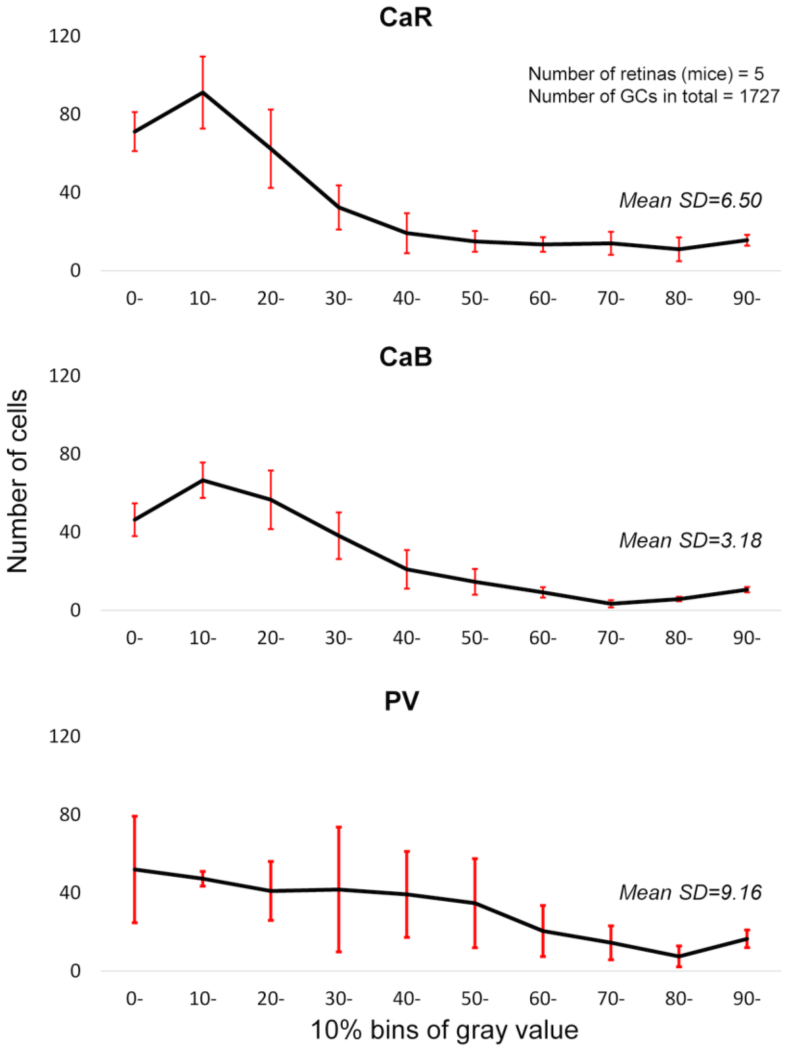

Figure S4: Variance between expression bins between individual retinal samples measured on the same dataset used in Figure 2. n=5 (nr. of mice); 1727 RGCs, 10% expression bins for CaR, CaB, PV.

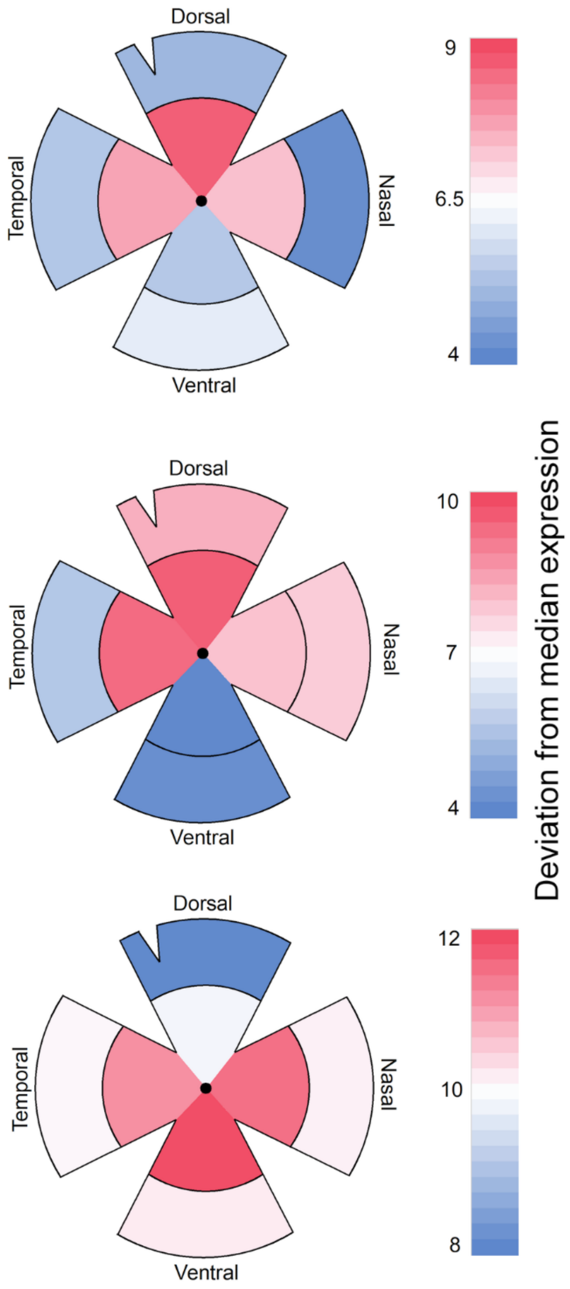

Figure S5: The deviation from median expression can be seen color-coded, representing cell numbers with higher expression levels with red, lower numbers with blue and white closest to the median. In all cases, except CaR and CaB ventral quadrant, show a stronger expression in the central part of each quadrant. Variance in CaR expression is observable between the center and periphery. In the case of CaB and PV, some variance could be detected, which is restricted only to the difference from the median.
